# Immune-Mediated Mechanisms of COVID-19 Neuropathology

**DOI:** 10.3389/fneur.2022.882905

**Published:** 2022-05-19

**Authors:** Cordelia Dunai, Ceryce Collie, Benedict D. Michael

**Affiliations:** ^1^Department of Clinical Infection Microbiology and Immunology, Institute of Infection, Veterinary and Ecological Sciences, University of Liverpool, Liverpool, United Kingdom; ^2^NIHR HPRU for Emerging and Zoonotic Infection, Liverpool, United Kingdom; ^3^The Walton Centre NHS Foundation Trust, Liverpool, United Kingdom

**Keywords:** COVID-19, immunology, neurological complications, cytokine release syndrome, autoantibodies

## Abstract

Although SARS-CoV-2 causes a respiratory viral infection, there is a large incidence of neurological complications occurring in COVID-19 patients. These range from headaches and loss of smell to encephalitis and strokes. Little is known about the likely diverse mechanisms causing these pathologies and there is a dire need to understand how to prevent and treat them. This review explores recent research from the perspective of investigating how the immune system could play a role in neurological complications, including cytokines, blood biomarkers, immune cells, and autoantibodies. We also discuss lessons learnt from animal models. Overall, we highlight two key points that have emerged from increasing evidence: (1) SARS-CoV-2 does not invade the brain in the majority of cases and so the associated neurological complications might arise from indirect effects, such as immune activation (2) although the immune system plays a critical role in controlling the virus, its dysregulation can cause pathology.

## Introduction

Neurological symptoms occur in approximately one-third of hospitalised COVID-19 patients (1). These range from symptoms of headaches, loss of taste and smell to seizures, encephalitis, and stroke. Despite the prevalence of neurological manifestations in COVID-19 patients, scientists have struggled to find consistent evidence of direct viral invasion in neuronal tissue and cerebrospinal fluid (CSF) that can be causally attributed to these symptoms (2, 3). An upregulated immune response appears to be a factor in many of the neuropathologies observed in COVID-19 patients, including dysregulated cytokines, inflammation, coagulation, and the development of antibodies against self-antigens (4). This review will focus on what is known and what is hypothesised about immune-mediated damage during post-acute sequelae to nervous system insult from COVID-19. However, it is outside of the scope of this review to address the likely significantly heterogenous mechanisms underlying long-COVID syndrome.

**Figure 1:**
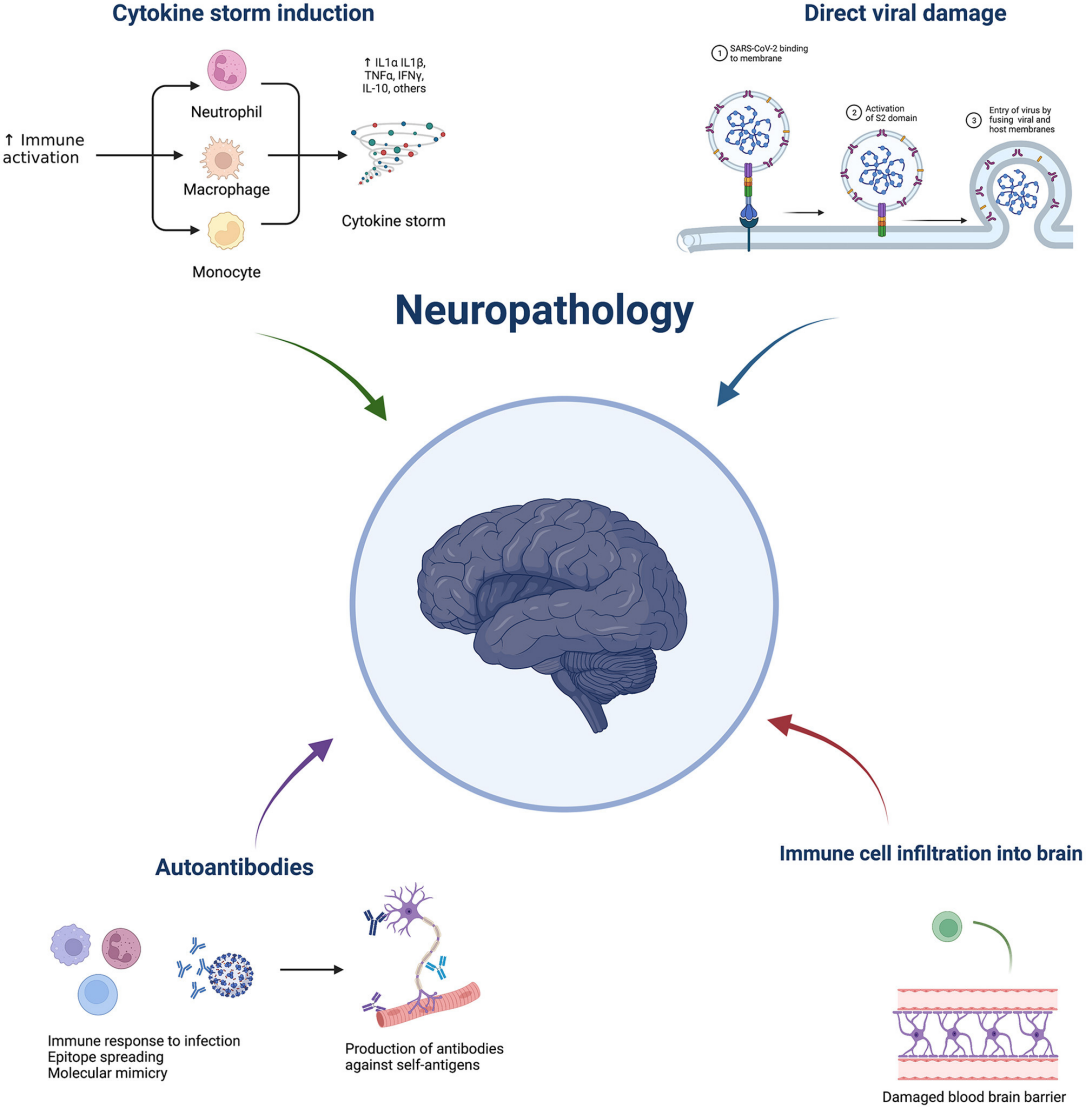
Overview of potential mechanisms underlying COVID-19 neuropathology. Made by BioRender.com.

A nationwide surveillance study, CoroNerve, found a higher frequency of neuropsychiatric symptoms in young adults vs. cerebrovascular events which had a higher frequency in adults over 60 years old (5). The incidence of neurological complications has decreased since the beginning of the pandemic and this could be due to the combination of better treatment regimens, use of immunosuppressants, and protection from vaccination. However, it is not purely the severity of disease that determines whether it is accompanied by neurological complications. Interestingly, neurological complications from respiratory viruses are not unique to COVID-19. Indeed, they have been reported with several pathogens, including influenza, tuberculosis, and SARS-CoV-1. Despite the significant suffering in the current pandemic, this is a unique opportunity to further understand these complications and discover prophylactic and effective treatment strategies. SARS-Cov-2 is not predominately neurotropic as a minority of autopsies show virus presence in the brain (6). Therefore, indirect viral effects are hypothesised to be responsible for neurological complications, such as a dysregulated immune response. This could include elevated cytokines, blood-brain barrier damage, immune infiltration, blood vessel inflammation, and blood vessel blockage leading to hypoxia, as well as immune-mediated tissue damage mediated by cells and/or autoantibodies. Neuroinflammation can broadly be grouped into central vs peripheral pathologies and subgroups within central include: cerebrovascular (e.g., stroke), encephalitis/encephalopathies, seizures, and movement disorders. Neuroinflammation following SARS-CoV-2 can cause short- or long-term pathology. This review aims to discuss the possible immune-mediated mechanisms behind pathologies and what has been learnt from animal models (Figure 1). Clinical features and neuroimaging have been described in detail and reviewed previously (7–9).

## Viral vs. Immune-Mediated Pathology

### Viral Damage and the Olfactory Route

Although the SARS-CoV-2 virus has been found in a minority of brains in autopsies, direct damage to the nervous system can occur and *in vitro*, brain organoids and neurons have been found to become infected by SARS-CoV-2 virus (10, 11). One possible route for the virus to the brain is through the nose—viral presence has been observed all the way up the path into the brain—in the olfactory mucosa, olfactory bulb, olfactory tubercle, oral mucosa, trigeminal ganglion, medulla oblongata, but only rarely in the cerebellum (6). A confounding variable in post-mortem studies is autolysis, so it is important to note that a study, which involved post-mortem bedside surgical tissue extraction, found no evidence of infection in the olfactory sensory neurons or olfactory bulb parenchyma (12). Direct or indirect alterations to the olfactory receptors are thought to be the cause of loss of smell observed in many COVID-19 cases. Other routes of the virus into the brain are *via* endothelial cells and *via* a Trojan horse approach within migrating immune cells. Once neuronal cells are infected, they can release pro-inflammatory cytokines and chemokines which in turn attract immune cells. Cytotoxic T cells have been found in some brain autopsies (13). Although this is an important anti-viral immune response, infiltration into the central nervous system and immune-mediated cytotoxicity can cause collateral tissue damage. A recent study found a novel mechanism by which SARS-CoV-2 directly damages the brain, through infection of endothelial cells which leads to their death and results in significant loss of capillaries, leading to hypoxia and blood-brain barrier damage (14).

### Cytokines and Chemokines and Other Immune Biomarkers

Cytokines and chemokines play an important role in the anti-viral response, but their dysregulation can lead to detrimental side effects, such as uncontrolled fever, delirium, liver, brain, and other tissue inflammation and damage. A lot has been learned about dysregulated immune function and successful treatments in the context of autoimmune diseases. For example, cytokine release syndrome observed with CAR T cell neurotoxicity can be treated with the rheumatoid arthritis drug, anti-IL-6R (15). The “usual suspects” of detrimental cytokine release syndrome are pro-inflammatory cytokines: IL-6, TNF, IFNγ as well as chemokines that act on neutrophils (IL-8) and monocytes (CCL2 and CCL5) and cytokines that stimulate granulocyte and monocyte production (GM-CSF) (4, 16). Although many cytokines and chemokines play roles in activating and attracting immune cells, it is promising that single-target inhibitors, such as anti-IL-6R, have shown efficacy in treating pulmonary COVID-19 (17, 18). Anti-TNF treatment has also been associated with reduced hospitalisation from COVID-19 (19).

Measurements of cerebrospinal fluid (CSF) offer important insight into the central nervous system (CNS). A key early study found elevations of brain injury markers neurofilament-light chain, T-tau, and glial fibrillary acidic protein and IL-8 in the CSF of SARS-CoV-2-associated encephalitis cases (20). A systematic review found that the minority of cases of CNS manifestations had viral presence and virus-specific antibodies in the CSF, but the majority had elevated brain injury markers and pro-inflammatory cytokines, such as IL-1β, IL-2, IL-6, IL-8, and TNF (21). The pathologies with the greatest incidence of viral presence were encephalitis and meningitis (21). The presence of inflammation in the absence of virus and virus-specific antibodies implicates indirect effects of the virus on neuropathology.

From a mouse model of herpes simplex virus, it is known that the IL-1α induction of CXCL1 production by astrocytes and neurons and subsequent neutrophil migration into the brain is an important pathway in blood-brain barrier breakdown (22). This remains to be investigated in SARS-CoV-2 infection, but neutrophils have been observed in brain infiltrates in humans (8). Another related target for reducing inflammation is the IL-1α and IL-1β receptors. Treatment with anakinra, an IL-1R antagonist, has shown a beneficial effect when compared to historical controls, with reduced need for mechanical ventilation and mortality in COVID-19 patients (23). In terms of a potential prognostic biomarker for COVID-19 patients, it has been found that levels of IL-6 were increased at admission for patients with neurological disorders (24). Many immune biomarkers have been associated with COVID-19 disease severity, including IL-6, IL-8, IL-10, IL-17A, IL-1RA, IFNγ, GM-CSF, HGF, CCL1, and CXCL13, however, specific roles in neuropathology have not been elucidated for all of them yet (4, 16, 25, 26). However, there has been a fascinating set of case studies published with in-depth clinical data and neuroimaging which report that neurological manifestations occurred at the same time as cytokine release syndrome (27). HGF, IL-10, and IL-1RA are very interesting as biomarkers of disease severity as they all have a protective role. HGF is involved in lung tissue repair, IL-10 is a regulatory cytokine and IL-1RA antagonises both IL-1α and IL-1β signalling. It is hypothesised that these are outstanding biomarkers of a regulatory response against inflammation and tissue damage in severe disease.

Complement components C3, C5a, and C5b have also been associated with disease severity (28). Although complement is an important part of the innate anti-viral response directly triggered by viral infection, hyperactivation can lead to detrimental coagulation, thrombosis, and endothelial cell injury. Clinical trials with drugs that block complement components are ongoing.

The broad immunosuppressant dexamethasone has shown efficacy in COVID-19 clinical trials, but importantly its protective effect was limited to patients requiring supplemental oxygen and in patients with less severe disease, it potentially had a detrimental effect (29). This highlights two important aspects: (1) there is a fine balance between suppressing the immune system to avoid detrimental effects and maintaining the anti-viral response and (2) more specific immune-suppressing treatments are needed.

## Vascular Complications

Hyperactivation of the immune system plays a key role in adverse events involving blockage of blood vessels and damage to blood vessel walls. Vascular complications, such as hypercoagulopathy, thrombosis, and endotheliitis, can cause a range of pathologies, including neuropathologies when the nervous system is affected. Notable neuropathologies involving these mechanisms are ischaemic and haemorrhagic stroke, cerebral venous sinus thrombosis, and intracerebral endotheliitis. Damage to the blood-brain barrier can start a vicious cycle of loss of protection from the virus and cellular infiltration, and result in autoantigen release in the context of inflammatory mediators.

Several biomarkers of blood clotting have been associated with severe COVID-19, including von Willebrand Factor, factor VII, and factor VIII (30). In a previously mentioned study, in addition to IL-6, D-dimers, which are formed during the coagulation cascade, had increased levels in patients with neurological disorders at the time of admission (24). In addition to vascular complications, cytokine release syndrome can further exacerbate damage to the blood-brain barrier and cause neurotoxicity.

## Autoantibodies

It is known from several autoimmune diseases that autoantibodies can play a role in damaging the nervous system. Indeed, the delayed presentation of some neurological complications from SARS-CoV-2 infection potentially implicates a long-lasting immune response driven by memory T cells being active and memory B cells producing antibodies. CNS autoantibodies have been reported in various clinical cases and evidence for their role in COVID-19 neurology is currently under investigation. Among the cerebral disorders reported as secondary to SARS-CoV-2 infection, antibodies directed against the NMDA-receptor have been detected extremely rarely in serum or CSF (31, 32). Additionally, there are several autoantibody-driven peripheral syndromes, such as Guillain-Barré syndrome (GBS) and Miller Fisher Syndrome (MFS), that have been associated with COVID-19.

It has been hypothesised that autoimmune complications may be related to molecular mimicry of SARS-CoV-2 proteins with human proteins. One study reported only one protein with high homology between the SARS-CoV-2 and human proteome, while other studies have reported several identical peptide sequences (31–33). Hexa-peptide and hepta-peptide sharing of SARS-CoV-2 spike glycoprotein with human proteins has been identified as a potential contributor to the occurrence of autoimmune disorders observed in COVID-19 patients (34). Of note, but not specific to SARS-CoV-2 or neuropathology, autoantibodies against immune proteins, such as interferons, have been detected during COVID-19 and could impair the anti-viral response leading to more severe disease (35).

As the immune system develops antibodies to viral antigens, shared epitopes on self-antigens could become targets and result in adverse effects (36). However, it is unknown how often autoantibodies are the drivers of pathology and not just transient indicators of tissue damage. Additional work has been done to demonstrate the molecular mimicry between SARS-CoV-2 and human proteins expressed in vagal nuclei and ganglia, which may account for the low vagal tone observed in some cases of COVID-19 (37). This study also found that neurons within the dorsal motor nucleus, nucleus ambiguous, nodose ganglion, and jugular ganglion can all present antigens with epitopes shared with SARS-CoV-2 antigens. More potential targets of SARS-CoV-2 molecular mimicry have been identified in the brain, such as neuron navigator protein 1, neuron navigator protein 3, and corticotropin-releasing factor receptor 2 (38). However, there appears to be a lack of clinical or pre-clinical data to show that these antigens are actual targets in neurological manifestations.

Bystander activation and epitope spreading may also explain some neuropathology in COVID-19 patients, particularly those presenting with cerebrovascular complications. The overreactive immune response creates a pro-inflammatory environment that may damage the blood-brain barrier, myelin sheath, and axonal membrane (39). As the damage of self-tissues continues, more self-antigens are released. This can potentially increase the activation of autoreactive T-cells and trigger chronic and progressive CNS degenerative disease pathology (38).

A range of autoantibodies has been identified in COVID-19 patients, but evidence of their role in the development of particular neurological autoimmune disorders has only been found for select autoantibodies. GBS has been of particular interest as it was the first documented neurological autoimmune disease to be implicated with COVID-19. This polyneuropathy is characterised by an autoimmune attack on peripheral nerve myelin and has been reported in various post-infectious complications associated with other pathogens, such as *Campylobacter jejuni*, Epstein-Barr virus, herpes simplex virus, and cytomegalovirus. It is hypothesised that pathogenic ganglioside epitopes stimulate the production of antibodies that cross-react with myelin antigens, leading to the observed neurological deficits (40). A systematic review of 73 GBS cases found median time from COVID-19 to onset of GBS symptoms was 14 days, but intriguingly, the majority of patients were negative for anti-ganglioside antibodies that are frequent in GBS and MFS (41). It is important to note that overall GBS incidence has been reduced during the COVID-19 pandemic (and associated infectious disease precautions) and there is no epidemiological correlation between the COVID-19 pandemic and GBS co-incidence (33).

One study of CSF and serum autoantibodies in patients with neurological manifestations identified anti-neuronal antibodies. Among the targeted antigens were intracellular and neuronal surface proteins as well as a number of undetermined epitopes (42). Another study found higher levels of anti-phosphatidylserine/prothrombin IgG in the COVID-neurological group compared to both control groups—non-neurological COVID and non-COVID hospitalised controls (43). More work with larger sample sizes and of people with a range of neurological disorders is needed to further elucidate exactly which antibodies are associated with different neuropathologies.

## Lessons from Animal Models

Similar pathology to human disease occurs in mammalian models of SARS-CoV-2 infection, mediated by infection of epithelial cells, elevated pro-inflammatory cytokines, and lung pathology, including acute respiratory distress syndrome.

There are two major strategies for infection in mice: (1) having the human ACE2 receptor (hACE2) expressed in mice either by transgenic expression (under the epithelial cell cytokeratin-18 promoter) or CRISPR/Cas9 knock-in of hACE2 (replacing mouse ACE2) or viral-vector-delivered hACE2 or (2) use of mouse-adapted viral strains (44). Interestingly, SARS-CoV-2 spreads to the mouse brain and is implicated as a cause of lethality which is quite different than human pathology (44–47). SARS-CoV-1 also showed dissemination to the brain (48). A recent study showed the ubiquity of SARS-CoV-2 infection in mice and delineated the rapid time course of neuroinvasion (49). This study also discovered that the Fc portion of antibodies, not just the binding regions, was important for the anti-viral response (49). This highlighted that NK cells, macrophages, and neutrophils play a role in clearing the virus dependent on FcR binding involved in antibody-dependent cytotoxicity and phagocytosis and emphasised the consideration needed for the Fc region when developing effective therapeutic antibodies.

One of the greatest advantages of studying mice is the ability to assess disease phenotype in genetic knockouts and/or use monoclonal antibodies to block mediators or deplete cell types. Insights have been made into modulators of disease severity. For example, we have learned that T cell depletion or lack of *Ifnar1* or *Stat1* results in worse pathology (50). Conversely, mice deficient in complement component 3 showed protection from SARS-CoV-1 disease (51).

It is troubling that new omicron variants are able to infect wild-type mice as animal reservoirs pose a risk of prolonging the pandemic (52). A growing list of mammalian species have been found to be infected by the virus and it is unknown exactly what adaptations make this possible and how transmission is occurring and whether transmission back to humans is common (53). Other mammalian species have been used to study similar respiratory symptoms, lung pathology, and neutralising antibodies, including hamsters and ferrets (54).

Arguably, the most translatable research has been with monkey models, from which we have learned about viral shedding, anti-viral treatments, vaccines, neutralising antibodies, convalescent plasma, and long-term immunity, all of which have made a huge impact on the pandemic. An advantage of studying African green monkeys and rhesus macaques is that, like humans, they show more severe pathology in aged individuals (54). Similar to humans, cytokines and other immune markers were raised during SARS-CoV-2 infection and the cause of death in aged monkeys was attributed to interstitial pneumonia involving haemorrhage and necrosis in the lungs (55). Interestingly, there has been a report of SARS-CoV-2 causing viral encephalitis in a rhesus macaque (56). Although there were no clinical neurological symptoms observed, one out of three infected rhesus macaques showed endothelial cell hypertrophy, endotheliitis, and vascular inflammation in the brain (56). This indicates that non-human primates might be the most relevant animal model for neurological complications.

## Discussion

The explosion of novel findings on the spectrum of neurological complications of COVID-19 is a testament to what a transformative time this is. However, a major limitation to understanding COVID-19 neuropathology is the dependence on post-mortem autopsies which represent severe disease frequently involving co-morbidities and/or immunosuppression. Nevertheless, post-mortem examination of brains has given us important insight into neutrophil and T cell infiltration and microglia activation following infection (57). Overall, large-scale and clinically comprehensive studies are needed to learn about the spectrum of neurological complications. With so many groups working towards understanding these phenomena and investigating ways to prevent and treat them, we are in a strong position to make rapid progress in addressing the many unanswered questions. It is becoming clear that the majority of neurological complications involve neuroinflammation and sometimes hypoxic-ischemic damage which are is not driven by direct SARS-CoV-2 infection, but indirect effects of anti-viral and inflammatory responses. The correlation of heightened immune response with severe disease indicates that host response factors could be responsible for pathology (4). Although the heterogenous pathologies likely have different mechanisms and therapeutic targets, it is promising that at least the pulmonary manifestations of COVID-19 are amenable to immunosuppressant treatment, such as dexamethasone and anti-IL-6R. In addition to the fast-acting innate immune response, the more delayed range of neurological complications, especially demyelinating disease, implicates that the adaptive immune response is likely involved in pathology in certain individuals. Less is known about how to specifically target autoimmune memory B and T cells without broadly and potentially detrimentally suppressing the immune system, such as with drugs like methotrexate and mycophenolate mofetil as used in primary autoimmune diseases. Potentially, synergistic treatments, such as a combination of anti-viral drugs, targeted immune suppression, and possibly neuroprotective agents, will prove most effective at reducing the neuropathogenesis of central and peripheral nervous system complications of SARS-CoV-2. Now is our first chance to answer these crucial questions.

## Author Contributions

All authors listed have made a substantial, direct, and intellectual contribution to the work and approved it for publication.

## Funding

BM was supported to conduct COVID-19 neuroscience research by the UKRI/MRC (MR/V03605X/1). BM was also supported for additional neurological inflammation research due to viral infection by grants from the MRC/UKRI (MR/V007181//1), MRC (MR/T028750/1), and Wellcome (ISSF201902/3). Open access publication fees were paid by University of Liverpool. We thank the NIHR BioResource for their support.

## Conflict of Interest

The authors declare that the research was conducted in the absence of any commercial or financial relationships that could be construed as a potential conflict of interest.

## Publisher's Note

All claims expressed in this article are solely those of the authors and do not necessarily represent those of their affiliated organizations, or those of the publisher, the editors and the reviewers. Any product that may be evaluated in this article, or claim that may be made by its manufacturer, is not guaranteed or endorsed by the publisher.

## References

[1] Mao L Jin H Wang M Hu Y Chen S He Q. Neurologic manifestations of hospitalised patients with coronavirus disease 2019 in Wuhan, China. JAMA Neurol. (2020) 77:683–90. 10.1001/jamaneurol.2020.1127 32275288 PMC7149362

[2] Destras G Bal A Escuret V Morfin F Lina B Josset L. Systematic SARS-CoV-2 screening in cerebrospinal fluid during the COVID-19 pandemic. Lancet Microbe. (2020) 1:e149. 10.1016/S2666-5247(20)30066-5 32835345 PMC7289579

[3] Jacob F Pather SR Huang W-K Zhang F Wong SZH Zhou H. Human pluripotent stem cell-derived neural cells and brain organoids reveal SARS-CoV-2 neurotropism predominates in choroid plexus epithelium. Cell Stem Cell. (2020) 27:937–950.e9. 10.1016/j.stem.2020.09.016 33010822 PMC7505550

[4] Lucas C Wong P Klein J Castro TBR Silva J Sundaram M. Longitudinal analyses reveal immunological misfiring in severe COVID-19. Nature. (2020) 584:463–9. 10.1038/s41586-020-2588-y 32717743 PMC7477538

[5] Varatharaj A Thomas N Ellul MA Davies NWS Pollak TA Tenorio EL. Neurological and neuropsychiatric complications of COVID-19 in 153 patients: a UK-wide surveillance study. Lancet Psychiatry. (2020) 7:875–82. 10.1016/S2215-0366(20)30287-X 32593341 PMC7316461

[6] Meinhardt J Radke J Dittmayer C Franz J Thomas C Mothes R. Olfactory transmucosal SARS-CoV-2 invasion as a port of central nervous system entry in individuals with COVID-19. Nat Neurosci. (2021) 24:168–75. 10.1038/s41593-020-00758-5 33257876

[7] Ellul MA Benjamin L Singh B Lant S Michael BD Easton A. Neurological associations of COVID-19. Lancet Neurol. (2020) 19:767–83. 10.1016/S1474-4422(20)30221-0 32622375 PMC7332267

[8] Maiese A Manetti AC Bosetti C Del Duca F La Russa R Frati P. SARS-CoV-2 and the brain: a review of the current knowledge on neuropathology in COVID-19. Brain Pathol. (2021) 31:e13013. 10.1111/bpa.13013 34390282 PMC8420197

[9] Sriwastava S Tandon M Podury S Prasad A Wen S Guthrie G. COVID-19 and neuroinflammation: a literature review of relevant neuroimaging and CSF markers in central nervous system inflammatory disorders from SARS-COV2. J Neurol. (2021) 28:4448–4478. 10.1007/s00415-021-10611-9 34009454 PMC8131883

[10] Song E Zhang C Israelow B Lu-Culligan A Prado AV Skriabine S. Neuroinvasion of SARS-CoV-2 in human and mouse brain. J Exp Med. (2021) 218:e20202135. 10.1084/jem.20202135 33433624 PMC7808299

[11] Chu H Chan JFW Yuen TTT Shuai H Yuan S Wang Y. Comparative tropism, replication kinetics, and cell damage profiling of SARS-CoV-2 and SARS-CoV with implications for clinical manifestations, transmissibility, and laboratory studies of COVID-19: an observational study. Lancet Microbe. 1:e14–e23. 10.1016/S2666-5247(20)30004-5 32835326 PMC7173822

[12] Khan M Yoo S-J Clijsters M Backaert W Vanstapel A Speleman K. Visualizing in deceased COVID-19 patients how SARS-CoV-2 attacks the respiratory and olfactory mucosae but spares the olfactory bulb. Cell. (2021) 184:5932–5949.e15. 10.1016/j.cell.2021.10.027 34798069 PMC8564600

[13] Matschke J Lütgehetmann M Hagel C Sperhake JP Schröder AS Edler C. Neuropathology of patients with COVID-19 in Germany: a post-mortem case series. Lancet Neurol. (2020) 19:919–29. 10.1016/S1474-4422(20)30308-2 33031735 PMC7535629

[14] Wenzel J Lampe J Müller-Fielitz H Schuster R Zille M Müller K. The SARS-CoV-2 main protease Mpro causes microvascular brain pathology by cleaving NEMO in brain endothelial cells. Nat Neurosci. (2021) 24:1522–33. 10.1038/s41593-021-00926-1 34675436 PMC8553622

[15] Morris EC Neelapu SS Giavridis T Sadelain M. Cytokine release syndrome and associated neurotoxicity in cancer immunotherapy. Nat Rev Immunol. (2022) 22:85–96. 10.1038/s41577-021-00547-6 34002066 PMC8127450

[16] Thwaites RS Sanchez Sevilla Uruchurtu A Siggins MK Liew F Russell CD Moore SC. Inflammatory profiles across the spectrum of disease reveal a distinct role for GM-CSF in severe COVID-19. Sci Immunol. (2021) 6:eabg9873. 10.1126/sciimmunol.abg9873 33692097 PMC8128298

[17] Gupta S Leaf DE. Tocilizumab in COVID-19: some clarity amid controversy. Lancet. (2021) 397:1599–601. 10.1016/S0140-6736(21)00712-1 33933194 PMC8084409

[18] Nowill AE de Campos-Lima PO. Immune response resetting as a novel strategy to overcome SARS-CoV-2-induced cytokine storm. J Immunol. (2020) 205:2566–75. 10.4049/jimmunol.2000892 32958687

[19] Gianfrancesco M Hyrich KL Al-Adely S Carmona L Danila MI Gossec L. Characteristics associated with hospitalisation for COVID-19 in people with rheumatic disease: data from the COVID-19 Global Rheumatology Alliance physician-reported registry. Ann Rheum Dis. (2020) 79:859–66. 10.1136/annrheumdis-2020-217871 32471903 PMC7299648

[20] Pilotto A Masciocchi S Volonghi I De Giuli V Caprioli F Mariotto S. Severe acute respiratory syndrome coronavirus 2 (SARS-CoV-2) encephalitis is a cytokine release syndrome: evidences from cerebrospinal fluid analyses. Clin Infect Dis Off Publ Infect Dis Soc Am. (2021) 73:e3019–26. 10.1093/cid/ciaa1933 33395482 PMC7799260

[21] Domingues RB Leite FBV de M Senne C. Cerebrospinal fluid analysis in patients with COVID-19-associated central nervous system manifestations: a systematic review. Arq Neuropsiquiatr. (2022) 2002:S0004-282X2022005004205. 10.1590/0004-282X-ANP-2021-0117 35239818 PMC9648929

[22] Michael BD Bricio-Moreno L Sorensen EW Miyabe Y Lian J Solomon T. Astrocyte- and neuron-derived CXCL1 drives neutrophil transmigration and blood-brain barrier permeability in viral encephalitis. Cell Rep. (2020) 32:108150. 10.1016/j.celrep.2020.108150 32937134 PMC7548103

[23] Huet T Beaussier H Voisin O Jouveshomme S Dauriat G Lazareth I. Anakinra for severe forms of COVID-19: a cohort study. Lancet Rheumatol. (2020) 2:e393–400. 10.1016/S2665-9913(20)30164-8 32835245 PMC7259909

[24] Frontera JA Sabadia S Lalchan R Fang T Flusty B Millar-Vernetti P. A prospective study of neurologic disorders in hospitalized patients with COVID-19 in New York city. Neurology. (2021) 96:e575–86. 10.1212/WNL.0000000000010979 33020166 PMC7905791

[25] Perreau M Suffiotti M Marques-Vidal P Wiedemann A Levy Y Laouénan C. The cytokines HGF and CXCL13 predict the severity and the mortality in COVID-19 patients. Nat Commun. (2021) 12:4888. 10.1038/s41467-021-25191-5 34373466 PMC8352963

[26] Li L Li J Gao M Fan H Wang Y Xu X. Interleukin-8 as a biomarker for disease prognosis of coronavirus disease-2019 patients. Front Immunol. (2020) 11:602395. 10.3389/fimmu.2020.602395 33488599 PMC7820901

[27] Perrin P Collongues N Baloglu S Bedo D Bassand X Lavaux T. Cytokine release syndrome-associated encephalopathy in patients with COVID-19. Eur J Neurol. (2021) 28:248–58. 10.1111/ene.14491 32853434 PMC7461405

[28] Afzali B Noris M Lambrecht BN Kemper C. The state of complement in COVID-19. Nat Rev Immunol. (2022) 22:77–84. 10.1038/s41577-021-00665-1 34912108 PMC8672651

[29] Group TRC. Dexamethasone in hospitalized patients with Covid-19. N Engl J Med. (2020) 384:693–704. 10.1056/NEJMoa2021436 32678530 PMC7383595

[30] Quinaglia T Shabani M Breder I Silber HA Lima JAC Sposito AC. Coronavirus disease-19: the multi-level, multi-faceted vasculopathy. Atherosclerosis. (2021) 322:39–50. 10.1016/j.atherosclerosis.2021.02.009 33706082 PMC7883684

[31] Dotan A Muller S Kanduc D David P Halpert G Shoenfeld Y. The SARS-CoV-2 as an instrumental trigger of autoimmunity. Autoimmun Rev. (2021) 20:102792. 10.1016/j.autrev.2021.102792 33610751 PMC7892316

[32] Kreye J Reincke SM Prüss H. Do cross-reactive antibodies cause neuropathology in COVID-19? Nat Rev Immunol. (2020) 20:645–6. 10.1038/s41577-020-00458-y 33024283 PMC7537977

[33] Keddie S Pakpoor J Mousele C Pipis M Machado PM Foster M. Epidemiological and cohort study finds no association between COVID-19 and Guillain-Barré syndrome. Brain. (2021) 144:682–93. 10.1093/brain/awaa433 33313649 PMC7799186

[34] Kanduc D Shoenfeld Y. Molecular mimicry between SARS-CoV-2 spike glycoprotein and mammalian proteomes: implications for the vaccine. Immunol Res. (2020) 68:310–3. 10.1007/s12026-020-09152-6 32946016 PMC7499017

[35] Wang EY Mao T Klein J Dai Y Huck JD Jaycox JR. Diverse functional autoantibodies in patients with COVID-19. Nature. (2021) 595:283–8. 10.1038/s41586-021-03631-y 34010947 PMC13130511

[36] Blank M Barzilai O Shoenfeld Y. Molecular mimicry and auto-immunity. Clin Rev Allergy Immunol. (2007) 32:111–8. 10.1007/BF02686087 17426366

[37] Marino Gammazza A Légaré S Lo Bosco G Fucarino A Angileri F Oliveri M. Molecular mimicry in the post-COVID-19 signs and symptoms of neurovegetative disorders? Lancet Microbe. (2021) 2:e94. 10.1016/S2666-5247(21)00033-1 35544159

[38] Gupta M Weaver DF. COVID-19 as a trigger of brain autoimmunity. ACS Chem Neurosci. (2021) 12:2558–61. 10.1021/acschemneuro.1c00403 34213312

[39] Magro C Mulvey JJ Berlin D Nuovo G Salvatore S Harp J. Complement associated microvascular injury and thrombosis in the pathogenesis of severe COVID-19 infection: a report of five cases. Transl Res. (2020) 220:1–13. 10.1016/j.trsl.2020.04.007 32299776 PMC7158248

[40] Hughes RA Cornblath DR. Guillain-Barré syndrome. Lancet. (2005) 366:1653–66. 10.1016/S0140-6736(05)67665-9 16271648

[41] Abu-Rumeileh S Abdelhak A Foschi M Tumani H Otto M. Guillain-Barré syndrome spectrum associated with COVID-19: an up-to-date systematic review of 73 cases. J Neurol. (2021) 268:1133–70. 10.1007/s00415-020-10124-x 32840686 PMC7445716

[42] Franke C Ferse C Kreye J Reincke SM Sanchez-Sendin E Rocco A. High frequency of cerebrospinal fluid autoantibodies in COVID-19 patients with neurological symptoms. Brain Behav Immun. (2021) 93:415–9. 10.1016/j.bbi.2020.12.022 33359380 PMC7834471

[43] Benjamin LA Paterson RW Moll R Pericleous C Brown R Mehta PR. Antiphospholipid antibodies and neurological manifestations in acute COVID-19: a single-centre cross-sectional study. EClinicalMedicine. (2021) 39:101070. 10.1016/j.eclinm.2021.101070 34401683 PMC8358233

[44] Leist SR Dinnon KH Schäfer A Tse LV Okuda K Hou YJ. A mouse-adapted SARS-CoV-2 induces acute lung injury and mortality in standard laboratory mice. Cell. (2020) 183:1070–1085.e12. 10.1016/j.cell.2020.09.050 33031744 PMC7510428

[45] Carossino M Montanaro P O'Connell A Kenney D Gertje H Grosz KA. Fatal neuroinvasion and SARS-CoV-2 tropism in K18-hACE2 mice is partially independent on hACE2 expression. bioRxiv. (2021). 10.1101/2021.01.13.425144 35336942 PMC8955233

[46] Golden JW Cline CR Zeng X Garrison AR Carey BD Mucker EM. Human angiotensin-converting enzyme 2 transgenic mice infected with SARS-CoV-2 develop severe and fatal respiratory disease. JCI Insight. (2020) 5:e142032. 10.1172/jci.insight.142032 32841215 PMC7566707

[47] Kumari P Rothan HA Natekar JP Stone S Pathak H Strate PG. Neuroinvasion and encephalitis following intranasal inoculation of SARS-CoV-2 in K18-hACE2 Mice. Viruses. (2021) 13:132. 10.3390/v13010132 33477869 PMC7832889

[48] Glass WG Subbarao K Murphy B Murphy PM. Mechanisms of host defense following severe acute respiratory syndrome-coronavirus (SARS-CoV) pulmonary infection of mice. J Immunol. (2004) 173:4030–9. 10.4049/jimmunol.173.6.4030 15356152

[49] Ullah I Prévost J Ladinsky M Stone H Lu M Anand SP. Live Imaging of SARS-CoV-2 Infection in Mice Reveals Neutralizing Antibodies Require Fc Function for Optimal Efficacy. Rochester, NY: Social Science Research Network (2021). 10.2139/ssrn.3817810 PMC8372518 34453881

[50] Sun J Zhuang Z Zheng J Li K Wong RL-Y Liu D. Generation of a broadly useful model for COVID-19 pathogenesis, vaccination, and treatment. Cell. (2020) 182:734-743.e5. 10.1016/j.cell.2020.06.010 32643603 PMC7284240

[51] Gralinski LE Sheahan TP Morrison TE Menachery VD Jensen K Leist SR. Complement activation contributes to severe acute respiratory syndrome coronavirus pathogenesis. mBio. (2018) 10.1128/mBio.01753-18 30301856 PMC6178621

[52] Stone S Rothan HA Natekar JP Kumari P Sharma S Pathak H. SARS-CoV-2 variants of concern infect the respiratory tract and induce inflammatory response in wild-type laboratory mice. Viruses. (2022) 14:27. 10.3390/v14010027 35062231 PMC8777867

[53] Sharun K Dhama K Pawde AM Gortázar C Tiwari R Bonilla-Aldana DK. SARS-CoV-2 in animals: potential for unknown reservoir hosts and public health implications. Vet Q. (2021) 41:181–201. 10.1080/01652176.2021.1921311 33892621 PMC8128218

[54] Shou S Liu M Yang Y Kang N Song Y Tan D. Animal models for COVID-19: hamsters, mouse, ferret, mink, tree shrew, and non-human primates. Front Microbiol. (2021) 12:626553. 10.3389/fmicb.2021.626553 34531831 PMC8438334

[55] Blair RV Vaccari M Doyle-Meyers LA Roy CJ Russell-Lodrigue K Fahlberg M. Acute respiratory distress in aged, SARS-CoV-2-infected african green monkeys but not rhesus macaques. Am J Pathol. (2021) 191:274–82. 10.1016/j.ajpath.2020.10.016 33171111 PMC7648506

[56] Choudhary S Kanevsky I Yildiz S Sellers RS Swanson KA Franks T. Modeling SARS-CoV-2: comparative pathology in rhesus macaque and golden syrian hamster models. Toxicol Pathol. (2022) 2022:01926233211072767. 10.1177/01926233211072767 35128980 PMC8819578

[57] Schurink B Roos E Radonic T Barbe E Bouman CSC de Boer HH. Viral presence and immunopathology in patients with lethal COVID-19: a prospective autopsy cohort study. Lancet Microbe. (2020) 1:e290–9. 10.1016/S2666-5247(20)30144-0 33015653 PMC7518879

